# Efficacy and safety of a cream containing panthenol, prebiotics, and probiotic lysate for improving sensitive skin symptoms

**DOI:** 10.1111/srt.13540

**Published:** 2024-01-07

**Authors:** Xianghua Zhang, Delphine Kerob, Zhongxing Zhang, Han Tao, Xiaofeng He, Yi Yi, Xiaofeng Fang, Wenna Wang, Andrew Steel

**Affiliations:** ^1^ L'Oreal Dermatological Beauty L'Oréal China Shanghai China; ^2^ La Roche‐Posay Laboratoire Pharmaceutique Levallois Perret La Roche‐Posay France; ^3^ Research and Innovation Center L'Oréal China Shanghai China

**Keywords:** panthenol, prebiotics, probiotics, sensitive skin, skin barrier function

## Abstract

**Background:**

Sensitive skin is a common condition affecting a significant proportion of the population, and there is a growing demand for effective and safe management.

**Aim:**

To evaluate the efficacy and safety of a cream containing panthenol, prebiotics, and probiotic lysate as an optimal care for facial sensitive skin.

**Methods:**

A total of 110 participants (64 in group A and 46 in group B) with facial sensitive skin applied the cream twice daily for 28 days. Group A evaluated their sensitive skin, product efficacy, and product use experience at D0 (15 min), D1, D14, and D28. In group B, skin barrier function‐related indicators were measured at baseline and on D1, D7, D14, and D28. Dermatologists evaluated tolerance for all participants.

**Results:**

After 28 days of use, in group A, 100% of participants reported mildness and comfort with product use. Participants demonstrated significant improvements in skin barrier function‐related indicators, including increased stratum corneum moisture content, reduced erythema index, elevated sebum content, decreased trans‐epidermal water loss, and diminished skin redness parameter a* value (all *p* < 0.05). Dermatologist evaluations revealed excellent tolerance among all participants.

**Conclusion:**

The panthenol‐enriched cream with prebiotics and probiotic lysate exhibited substantial clinical efficacy in ameliorating facial sensitive skin conditions, coupled with a high safety profile.

## 1 INTRODUCTION

Sensitive skin is a multifaceted condition characterized by hyperreactivity to environmental factors. Symptoms can include itching, burning, pain, erythema, skin dryness, and scaling.^1^ Although not completely understood, the pathogenesis of sensitive skin is thought to involve an interplay of impaired skin barrier function, alterations in the skin microbiome, heightened neurosensory responses, and a tendency towards inflammation.^2^

The skin barrier plays a pivotal role in maintaining skin integrity by preventing excessive water loss and impeding the entry of potential irritants and allergens. Key indicators of skin barrier function include stratum corneum hydration, transepidermal water loss (TEWL), and sebum content. Impairments in the skin barrier can lead to increased susceptibility to environmental irritants and the worsening of sensitive skin symptoms.^3^ Additionally, recent studies have underscored the influence of the skin's microbiome on skin health and its possible contribution to the pathophysiology of sensitive skin.^4^ A balanced skin microbiome is crucial for proper barrier function, immune modulation, and the prevention of pathogenic colonization. Imbalances in the skin microbiome, characterized by a shift in the composition of resident bacteria such as *Cutibacterium acnes* (*C. acnes*), can lead to inflammatory responses and pruritus associated with sensitive skin.^5^

Various topical ingredients have been used to alleviate sensitive skin symptoms, with varying degrees of effectiveness. Notably, panthenol, prebiotics, and probiotic lysates have been explored for their potential benefits in managing sensitive skin. Panthenol, a provitamin B5 derivative, is recognized for its moisturizing, soothing, and skin barrier‐enhancing properties.^6^ Prebiotics, non‐digestible fibers that promote the growth of beneficial bacteria, and probiotic lysates, such as Aqua Posae Filiformis (APF), derived from the cell walls of probiotic bacteria, have been suggested to help restore skin microbiome balance, modulate immune responses, and reduce inflammation.^7^, ^8^, ^9^

This study aims to evaluate the efficacy and safety of a cream composed of panthenol, a blend of prebiotics, and probiotic lysate in improving symptoms of facial sensitive skin. The intended mechanisms of action include barrier repair, restoration of skin microbiome balance, immune regulation, and reduction of inflammation. Our study involved 110 subjects with facial sensitive skin, and the results showed promising efficacy. Given the complex nature of sensitive skin, understanding the underlying mechanisms and assessing the effectiveness of this combination treatment could offer valuable insights for developing targeted and personalized skincare strategies for individuals with sensitive skin.

## 2 METHODS

### 2.1 Study design and subjects

This single‐center, open‐label study enrolled Chinese male and female subjects aged 18–45 years with facial sensitive skin in Shanghai. The study was conducted by SGS‐CSTC Standards Technical Services (Shanghai) Co., Ltd., a globally independent testing, and certification company (with reference number: SHCPCH220705705 and SHCPCH221008564, SGS ethics committee for clinical research No. 2023019).

For the duration of the study, the external environmental conditions were recorded. Group A conducted their study in the summer, from July 13 to August 18, 2022, with Shanghai's temperatures ranging between 25°C and 38°C. Conversely, Group B's study period spanned the late autumn and early winter, from November 28, 2022, to January 3, 2023, with temperatures varying between −2°C and 15°C. Despite the distinct outdoor environmental conditions during the two study periods, all clinical tests were performed in a controlled laboratory environment, where the temperature was maintained at 21 ± 2°C and humidity at 50 ± 10%.

Inclusion criteria were (1) 18–45 years of age; (2) good general health; (3) normal to dry skin with facial sensitive skin; (4) self‐assessed sensitive skin; (5) sensitive skin screened by Lactic Acid Tingling Test, which is a validated method employed to distinguish individuals with sensitive skin, characterized by increased reactions such as stinging and itching, but not necessarily burning or erythema^10^; (6) Mandarin fluency; (7) no facial treatments in the past 6 months; and (8) cooperation with study requirements.

Key exclusion criteria were (1) allergies to facial skincare products; (2) facial conditions that may influence the test; (3) breastfeeding or pregnancy; (4) history of skin cancer; (5) major organ diseases; (6) psychological or mental diseases; (7) Herpes Simplex; (8) prescription acne medication within the past 3–6 months; (9) prescription‐strength skin‐lightening products within 3 months; (10) OTC skin‐lightening products within 2 weeks; (11) pre‐existing dermatologic conditions; (12) observable facial conditions affecting test results; (13) immunosuppression; (14) corticosteroid use within 4 weeks; (15) uncontrolled diseases; and (16) recent long‐term medication.

### 2.2 Intervention

Both groups treated their faces with the test cream, with ingredients including panthenol, TRIBIOMA (ECOSKIN + mannose), APF, hydroxyproline, zinc gluconate, manganese gluconate, hyaluronic acid, and glycerin, which was applied twice per day on the entire face for a duration of 28 days. The cream was applied post‐cleansing, with a cleanser provided by the sponsor, and replaced participants' regular cream products for the duration of the study.

### 2.3 Clinical assessment

During the study, dermatologists conducted a comprehensive clinical assessment of participants' skin conditions in both groups at several time points: prior to product application, 15 min post‐application, 1 day, 14 days, and 28 days after continuous use. The assessment encompassed both objective and subjective parameters. Objective evaluations included the presence and severity of erythema, edema, dryness or desquamation, peeling, and other skin reactions. Subjective evaluations, on the other hand, focused on the participants' perception of sensations such as burning, stinging, itching, tightness, tingling, and any other notable experiences. Each parameter was rated on a four‐point scale, ranging from 0 (none) to 3 (severe).

### 2.4 Instrumental assessment

Participants underwent a series of instrumental assessments, including VISIA‐CR imaging procedures and bioinstrumentation measurements. Prior to imaging, subjects ensured their faces were clean, free of makeup, and void of any jewelry that could interfere with the photographic area. Each participant was provided with a black matte headband and a black matte cloth to prevent any reflection or light interference. During the imaging process, subjects were carefully positioned with neutral expressions and gently closed eyes.

Digital images of each subject's face (including left, center, and right views) were captured using the VISIA‐CR imaging system (Canfield Imaging Systems, Fairfield, New Jersey, USA). The analysis specifically focused on the Area of Interest (AOI) on either the left or right side of the cheeks, according to a predetermined randomization list for each subject's RBX‐Red lighting mode images captured by VISIA CR. Skin tone a* values were subsequently analyzed using the IPP image analysis software.

For bioinstrumentation measurements, a series of specialized devices were used. The Mexameter MX 18 (Courage + Khazaka electronic GmbH, Köln, Germany) was utilized to precisely determine the content of melanin and erythema, the primary constituents of skin color. Triplicate Mexameter measurements were specifically taken on the center of each subject's right or left cheek. The measurement location was at the intersection of lines extending down from the outer corner of the eye and horizontally across the bottom of the nose.

Transepidermal water loss (TEWL), a key indicator of skin barrier function, was assessed using the Tewameter TM Hex (Courage + Khazaka electronic GmbH, Köln, Germany). For each measurement, a single Tewameter reading was taken at the center of each subject's right or left cheek. The specific measurement location was determined by the intersection of lines extending down from the middle of the eye and horizontally across the middle of the nose. A decrease in TEWL values reflects an improvement in the barrier properties of the skin.

Skin hydration effects were assessed with the Corneometer CM 825 (Courage + Khazaka electronic GmbH, Köln, Germany), which employs an electrical capacitance method to measure the moisture content in the stratum corneum. Triplicate measurements were specifically taken at the center of each subject's right or left cheek. The measurement location was chosen at the intersection of lines extending down from the outer corner of the eye and horizontally across the bottom of the nose. This location was selected to be on the opposite side of the face from where Tewameter measurements were conducted. An increase in measurement values indicates an improvement in skin hydration.

Lastly, sebum quantity on the skin was measured using the Sebumeter SM 815 (Courage + Khazaka electronic GmbH, Köln, Germany), which employs a photometric method, where the transparency of a synthetic material (sebutape) upon contact with sebum is measured to calculate the amount of sebum on the skin surface. Triplicate measurements were specifically taken on adjacent, non‐overlapping sites at the center of each subject's forehead. To ensure consistency, a transparent positioning film was used by clinic staff to mark and track the three measurement sites across all time points. A decrease in test values suggests a decrease in skin sebum.

### 2.5 Self‐assessment

Subjects conducted self‐assessments: (1) Sensitive skin self‐assessments were carried out before use, and at D0 (15 min), D1, D14, and D28 after use, using a 10‐point scale where higher scores indicated more severe subjective symptoms. (2) At D0 (15 min), D1, D14, and D28 after use, subjects evaluated product efficacy on a scale of 1–5, with satisfaction being the percentage of participants scoring above 3. (3) After 28 days, subjects assessed their experience with the product.^11^

### 2.6 Statistical analysis

Statistical analysis involved providing a descriptive summary for tolerance parameters and various evaluation parameters. Summary statistics included the mean, median, standard deviation (SD), and minimum and maximum values. Replicate measurements were averaged before analysis, and mean changes from baseline were estimated. The null hypothesis was tested using appropriate methods. Percent mean changes and the percent of subjects improved or worsened were calculated for each parameter. The subjective evaluation scores were recognized as non‐parametric data, and thus, only medians were reported instead of averages. All statistical tests were two‐sided at significance level alpha = 0.05 unless specified otherwise. *p* values were reported to 3 decimal places (0.000). No multiple testing corrections were considered in the study. Statistical analyses were performed using SPSS software version 28.0.

## 3 RESULTS

### 3.1 Baseline characteristics of the participants

In this study, we initially screened 110 subjects. Of these, 64 subjects were allocated to the self‐assessment group (Group A), and 46 subjects were assigned to the instrumental assessment group (Group B). Within Group B, 42 subjects completed the study. The participants in Group A had a mean age of 31.72 years (SD = 7.64), ranging from 18 to 45 years. For Group B, the mean age was 31.88 years (SD = 6.81), with ages ranging from 20 to 45 years. However, in presenting our results, we have now unified the groups into a single study cohort, as the data from the two groups were not intended for comparative analysis. Instead, the objective was to evaluate the product's performance from both subjective and objective perspectives (Table 1).

**TABLE 1:** Demographic characteristics of participants in two different assessment groups.

Group	Number of participants	Mean age (years)	Age range (years)
Group A (Self‐assessment)	64 (22 male)	31.72	18–45
Group B (Instrumental assessment)	42 (1 male)	31.88	20–45

### 3.2 Treatment efficacy

The participants self‐assessed their sensitive skin symptoms using a 10‐point scale, with the results shown in Table 2. Immediately after 15 min of product use, participants reported significant reductions in skin irritation (41.92%), tingling sensation (92.75%), burning sensation (74.42%), heat sensation (81.25%), tightness (50.65%), itching (87.60%), pain (94.59%), and significant improvements in other discomforts. After 28 days of continuous use, all sensitive skin symptoms showed sustained and significant improvements compared to baseline (*p* < 0.05).

**TABLE 2:** Results of self‐assessment over various time points (D0, T15min, D1, D14, D28).

Parameter	D0 (Baseline)	T15min	D1	D14	D28
Skin irritation	2.61	1.52	0.95	0.38	0.09
Tingling sensation	1.08	0.08	0.05	0	0
Burning sensation	0.67	0.17	0	0	0
Heat/Hot flush	1	0.19	0.16	0.02	0.02
Tightness	1.2	0.59	0.38	0.2	0.06
Itchiness	1.89	0.23	0.06	0.03	0
Pain	0.58	0.03	0	0.06	0
General discomfort	1.45	0.72	0.17	0.03	0.02
Hot flashes	1.72	0.78	0.11	0	0
Redness	2.39	1.63	0.8	0.27	0.02

**FIGURE 1:**
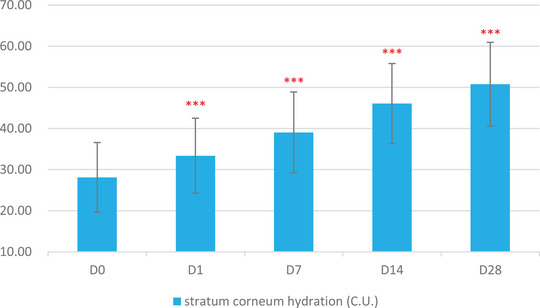
Changes in stratum corneum hydration over the course of the study.

**FIGURE 2:**
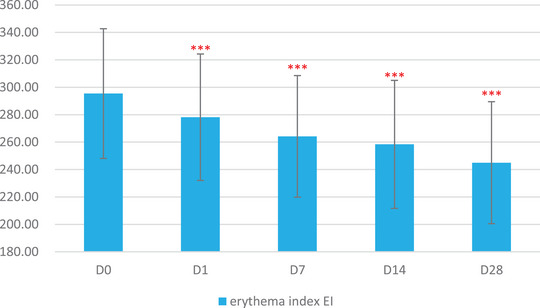
Changes in erythema index (EI) over the course of the study.

**FIGURE 3:**
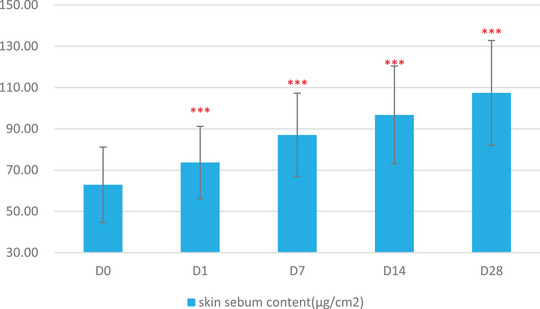
Changes in sebum content over the course of the study.

**FIGURE 4:**
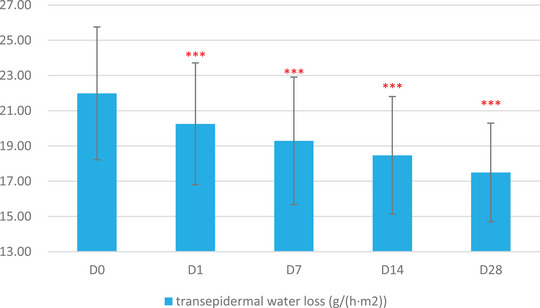
Changes in transepidermal water loss (TEWL) over the course of the study.

**FIGURE 5:**
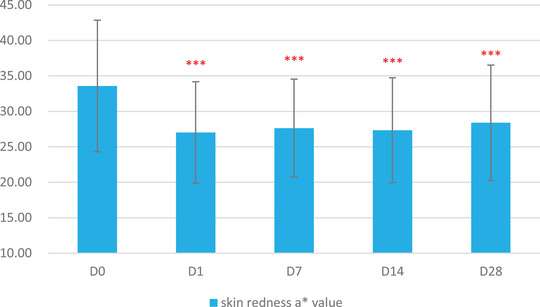
Changes in skin redness (a* value) over the course of the study.

**FIGURE 6:**
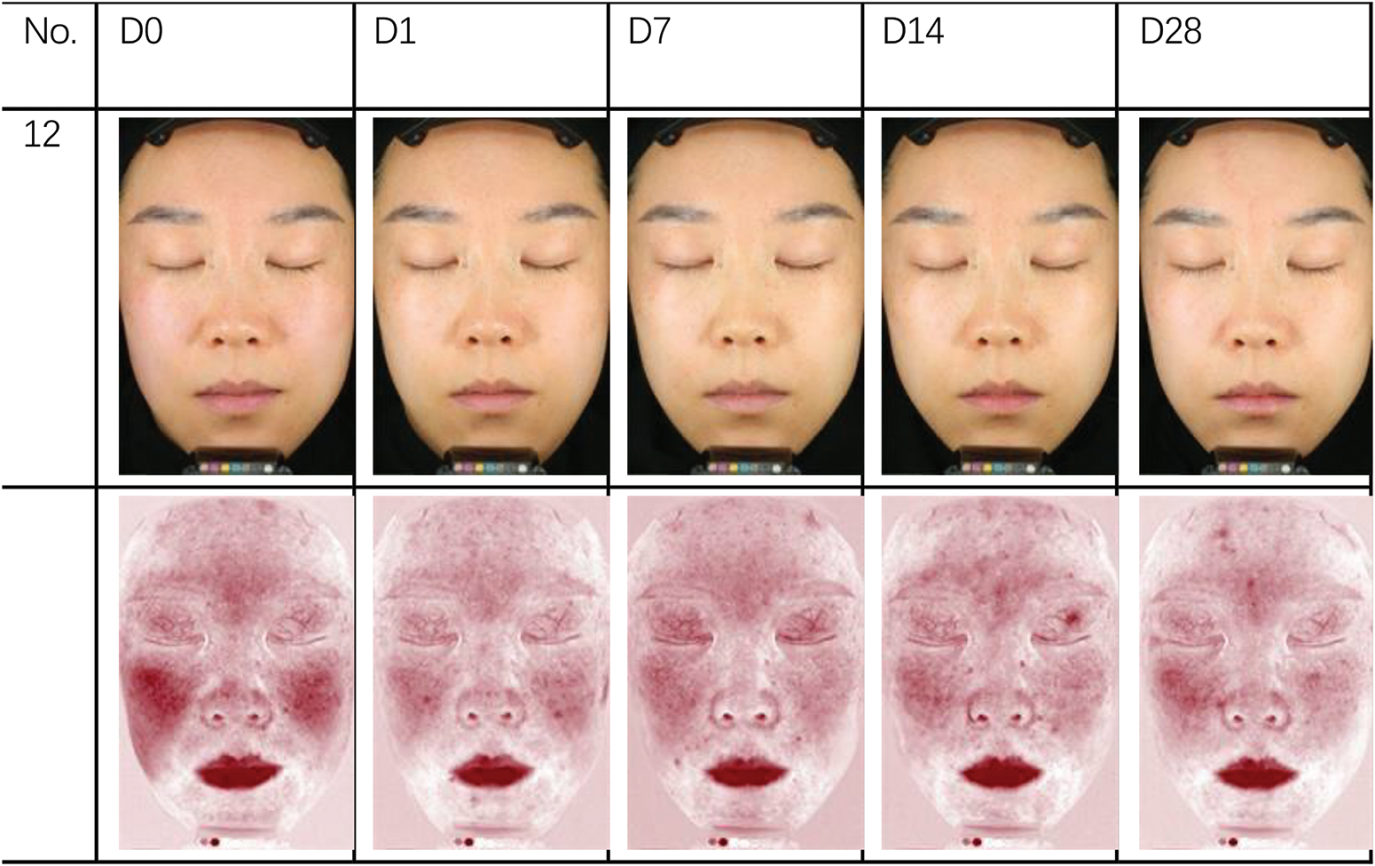
Changes in VISIA images for an average case over the course of the study.

The skin physiological parameters of Group B participants showed significant improvements compared to baseline (D0). For stratum corneum hydration, there was a significant increase of 18.72% at D1, 38.86% at D7, 63.79% at D14, and 80.60% at D28 (Figure 1 and Table 3). For erythema index (EI), significant reductions were observed at 5.84% on D1, 10.57% on D7, 12.55% on D14, and 17.06% on D28 (Figure 2 and Table 4). Sebum content showed significant increases of 17.14% on D1, 38.47% on D7, 53.87% on D14, and 70.85% on D28 (Figure 3 and Table 5). For transepidermal water loss (TEWL), significant decreases were observed at 7.88% on D1, 12.29% on D7, 16.04% on D14, and 20.42% on D28 (Figure 4 and Table 6). Lastly, skin redness (a* value) exhibited significant reductions of 19.50% on D1, 17.68% on D7, 18.55% on D14, and 15.46% on D28 (Figure 5 and Table 7) (*p* < 0.05 for all above data), an example of VISIA images of an average case shown in Figure 6.

**TABLE 3:** Results of skin redness area a * value over various time points (Baseline, D1, W1, W2, W4).

	Baseline	D1	W1	W2	W4
Mean	33.58	27.03	27.64	27.35	28.39
SD	9.28	7.15	6.91	7.39	8.14
Maximum	49.49	45.98	43.15	48.75	44.01
Minimum	15.04	14.37	14.13	14.93	14.30
Median	31.11	27.36	26.78	25.23	26.55
Rate of change	/	−19.50%	−17.68%	−18.55%	−15.46%
Significant *p* value compared with baseline	/	<0.001	<0.001	<0.001	<0.001

**TABLE 4:** Results of Sebumeter measurements over various time points (Baseline, D1, W1, W2, W4).

	Baseline	D1	W1	W2	W4
Mean	62.90	73.67	87.10	96.78	107.46
SD	18.27	17.54	20.18	23.69	25.40
Maximum	93.67	99.33	140.33	147.67	162.67
Minimum	21.00	25.00	37.67	37.67	52.00
Median	68.67	78.83	89.33	103.00	107.83
Rate of change	/	17.14%	38.47%	53.87%	70.85%
Significant *p* value compared with baseline	/	<0.001	<0.001	<0.001	<0.001

**TABLE 5:** Results of Sebumeter measurements over various time points (Baseline, D1, W1, W2, W4).

	Baseline	D1	W1	W2	W4
Mean	21.99	20.26	19.29	18.47	17.50
SD	3.77	3.46	3.62	3.34	2.80
Maximum	31.59	27.91	29.90	27.22	25.46
Minimum	15.63	14.05	13.21	13.37	12.22
Median	21.85	20.68	18.59	17.82	16.92
Rate of change	/	−7.88%	−12.29%	−16.04%	−20.42%
Significant *p* value compared with baseline	/	<0.001	<0.001	<0.001	<0.001

**TABLE 6:** Results of Corneometer measurements over various time points (Baseline, D1, W1, W2, W4).

	Baseline	D1	W1	W2	W4
Mean	28.12	33.38	39.05	46.06	50.78
SD	8.47	9.12	9.84	9.74	10.18
Maximum	46.53	51.03	55.47	68.50	69.50
Minimum	10.97	13.13	21.17	28.13	27.00
Median	26.65	33.97	39.28	47.27	52.93
Rate of change	/	18.72%	38.86%	63.79%	80.60%
Significant *p* value compared with baseline	/	<0.001	<0.001	<0.001	<0.001

**TABLE 7:** Results of Mexameter measurements over various time points (Baseline, D1, W1, W2, W4).

	Baseline	D1	W1	W2	W4
Mean	295.40	278.15	264.19	258.34	245.00
SD	47.30	46.10	44.35	46.67	44.48
Maximum	443.33	416.67	411.00	401.67	348.33
Minimum	204.33	198.00	196.33	180.00	144.67
Median	292.83	281.17	255.83	249.00	234.83
Rate of change	/	−5.84%	−10.57%	−12.55%	−17.06%
Significant *p* value compared with baseline	/	<0.001	<0.001	<0.001	<0.001

The tolerance of the cream was evaluated in two groups of subjects before use, as well as at 15 min, 1 day, 7 days, 14 days, and 28 days after use. The results showed that after 28 days of product use, symptoms, and signs of sensitive skin, such as burning sensation, stinging sensation, itching, tightness, tingling sensation, and skin redness, were alleviated to varying degrees in the subjects. Throughout the entire testing process, the subjects exhibited good tolerance with no adverse events reported.

### 3.3 Self‐assessment outcomes

Participants evaluated the effectiveness of the product, and the results demonstrated that, after 28 days of use, 100% of the participants were satisfied with various aspects of the product. They believed that the product not only improved their skin's overall appearance but also provided a comfortable and pleasant experience during use, with a gentle and agreeable texture. The participants expressed their willingness to continue using the product and showed purchase intentions.

## 4 DISCUSSION

This open‐label study aimed to investigate the performance and efficacy of a novel skincare product specifically formulated for individuals with sensitive skin. The study employed a two‐pronged approach to provide both subjective user experiences and objective technical measurements of the product's efficacy. The rigorous control of testing conditions was critical to ensure that the outcomes of the study were not influenced by external environmental factors, thus allowing for a clear assessment of the product's efficacy on facial sensitive skin. The initial self‐assessment served as a preliminary investigation into the real‐world performance of the product and its perceived benefits from a user perspective. Following these promising results, the study validated these subjective experiences with instrumental assessments, which generated quantifiable data on the product's effects.

Key ingredients in the product, including panthenol, a blend of prebiotics, and probiotic lysate, were selected to improve skin barrier function, maintain a balanced microbiota, provide essential nutrients for skin repair and regeneration, and deliver potent moisturizing and anti‐inflammatory effects.

Panthenol is known for its ability to promote skin hydration due to its hydrophilic nature, which can increase the water content in the stratum corneum and protect the epithelial tissue while promoting cell proliferation.^12^ Studies have shown that panthenol can enhance epidermal cell proliferation and differentiation while increasing epidermal lipid synthesis, thereby improving the lipid content in the epidermis.^13^ These properties can help to strengthen the skin's barrier function. Furthermore, panthenol has anti‐inflammatory properties that can help reduce inflammation and promote the symbiosis of probiotics in the skin microbiota.^14^

The skin microbiota plays a crucial role in maintaining the balance and integrity of the skin barrier. In cosmetic formulations, the focus is often on improving skin health by incorporating prebiotics, probiotics, and lysates.^15^ APF is a biologically active ingredient derived from the lysate of Vitreoscilla Filiformis (VF) bacteria, which is grown in La Roche‐Posay Thermal Spring Water enriched with rare elements such as Selenium and Strontium. These elements enhance the activity of VF, ultimately resulting in the creation of the “Super VF” lysate known as APF. APF can help modulate immune function by optimizing and regulating cell function and help the skin barrier achieve better recovery and immune resistance.^16^ Clinical studies have shown that 1% APF has a significant positive effect on improving the skin's microbial composition.^17^ The use of skincare products containing APF has been shown to increase skin microbiota diversity and reduce the abundance of Staphylococcus.^18^

Ecoskin, a plant extract containing α‐glucan oligosaccharide, inulin, maltodextrin, and Lactobacillus, is an inactivated prebiotic‐probiotic complex that can serve as a matrix for bacteria, helping to maintain skin microbiota balance while strengthening the skin's natural barrier. Mannose is a monosaccharide with prebiotic effects and immune‐stimulating properties, which can improve skin immunity while providing nutrients for probiotics, thereby enhancing skin barrier function.^19^

Hydroxyproline possesses excellent moisturizing and anti‐inflammatory effects, exerting its action by inhibiting the activation of nuclear factor kappa B (NF‐κB) and Toll‐like receptor 2 (TLR2)‐mediated signaling pathways.^20^ Hyaluronic acid is a naturally occurring component in the human body, with more than half of it found in the skin. It is considered an ideal natural moisturizing factor due to its exceptional water‐binding capacity.^21^ Other research has also shown that moisturizers can provide hydration and prevent water loss from the skin while positively affecting the epidermal microbiota and improving skin barrier function, similar to prebiotic effects.^22^

In this study, significant alignment was observed between subjective self‐assessments and objective instrumental data, providing a robust validation of the product's efficacy. Participants reported notable alleviation in a spectrum of sensitive skin symptoms—ranging from irritation and tightness to itching and redness. These self‐assessments were not merely anecdotal but were substantiated by a suite of bioinstrumental measurements. For instance, improvements in stratum corneum hydration could be attributed to the hydrophilic properties of panthenol, a key ingredient in the product formulation known for its moisture‐binding capabilities.^12^ The observed reductions in erythema index and skin redness may be linked to the anti‐inflammatory effects of panthenol and the microbiota‐balancing actions of APF.^16^, ^17^ Furthermore, the decrease in transepidermal water loss—an indicator of enhanced skin barrier function—suggests that the blend of prebiotics and probiotic lysate in the product effectively fortified the skin's natural defenses. Concurrent with this improvement in barrier function, we observed a significant increase in sebum levels from an average of 60 μg/cm^2^ at baseline to 110 μg/cm^2^ on Day 28. The application of the product appears to have supported the restoration of the skin's barrier integrity, especially for the participants with normal to dry sensitive skin types, which in turn may have normalized the function of the sebaceous glands, leading to increased sebum production. These measurable improvements in skin barrier markers lend scientific credence to the subjective experiences of reduced irritation and discomfort reported by the participants.

Despite these promising results, some limitations need to be acknowledged. The study period was relatively short, and longer‐term studies would be needed to assess the sustained effects of the product on skin barrier function and sensitive skin symptoms. Furthermore, the study did not include a placebo or control group, which could potentially introduce bias in the interpretation of the results. Future research could benefit from incorporating a double‐blind, placebo‐controlled design to further validate the efficacy of the product.

Moreover, the study focused primarily on the assessment of clinical outcomes, and the impact of the product on the skin microbiota was not directly evaluated. Future research could include an analysis of changes in the skin microbiota composition, diversity, and function to better understand the relationship between the product's ingredients and the observed clinical improvements. This could help elucidate the mechanisms by which the product enhances skin barrier function and alleviates sensitive skin symptoms.

It was noteworthy that there were differing trends in skin redness reduction between Group A and Group B, which was due to different assessment methods and environmental factors. Group A's self‐reported decrease over 28 days may reflect a heightened awareness and psychological influence on perceived skin condition. For Group B, the initial reduction in objective redness measurements followed by a slight increase could be influenced by the colder and drier autumn and winter climate, as sensitive skin is known to react more severely under such conditions.^23^

Despite the limitations, this study provides evidence for the efficacy and safety of the skincare product in improving skin barrier function and alleviating sensitive skin symptoms. The product's unique combination of ingredients, including panthenol, TRIBIOMA (ECOSKIN + mannose), APF, hydroxyproline, zinc gluconate, manganese gluconate, hyaluronic acid, and glycerin, appear to work synergistically to provide a comprehensive approach to addressing the complex needs of sensitive skin. The good tolerability and absence of adverse events reported in this study further support the suitability of the product for long‐term use in individuals with sensitive skin.

In conclusion, the skincare product, with its unique combination of key ingredients, demonstrates potential to improve skin barrier function and alleviate sensitive skin symptoms, offering a comprehensive approach to addressing the complex needs of individuals with sensitive skin. The significant improvements in hydration, sebum production, erythema index, transepidermal water loss, and self‐reported symptoms, coupled with the absence of reported adverse events, make the product a promising candidate for individuals with sensitive skin.

## CONFLICT OF INTEREST STATEMENT

No conflict of interest was reported by the authors.

## Data Availability

The data that support the findings of this study are available on request from the corresponding author. The data are not publicly available due to privacy or ethical restrictions.
