# Correction to “Efficacy and safety of a cream containing panthenol, prebiotics, and probiotic lysate for improving sensitive skin symptoms”

**DOI:** 10.1111/srt.13866

**Published:** 2024-07-19

**Authors:** Xianghua Zhang, Delphine Kerob, Zhongxing Zhang, Han Tao, Xiaofeng He, Yi Yi, Hui Xu, Wenna Wang, Steel Andrew

**Affiliations:** ^1^ L'Oreal Dermatological Beauty L'Oréal China Shanghai China; ^2^ La Roche‐Posay Laboratoire Pharmaceutique Levallois Perret France; ^3^ Research and Innovation Center L'Oréal China Shanghai China

Zhang X, Kerob D, Zhang Z, et al. Efficacy and safety of a cream containing panthenol, prebiotics, and probiotic lysate for improving sensitive skin symptoms. Skin Res Technol. 2024;30:e13540

In paragraph 1 of the “Intervention” section, the text “hydroxyproline” was incorrect. This should have read: “madecassoside”.

In paragraph 6 of the “DISCUSSION” section, the text “Hydroxyproline” was incorrect. This should have read: “Madecassoside”.

In paragraph 11 of the “DISCUSSION” section, the text “hydroxyproline” was incorrect. This should have read: “madecassoside”.

We apologize for these errors.

